# Pulmonary Leiomyosarcoma: A Diagnostic Challenge

**DOI:** 10.7759/cureus.96267

**Published:** 2025-11-06

**Authors:** Shazia Sultana, Dhanasekar Thangaswamy

**Affiliations:** 1 Department of Pulmonology, Sri Ramachandra Institute of Higher Education and Research, Chennai, IND

**Keywords:** doxorubicin, immunohistochemistry, pazopanib, primary lung sarcoma, pulmonary leiomyosarcoma, spindle cell tumor

## Abstract

Primary pulmonary leiomyosarcoma (PPL) is an extremely rare form of lung cancer, accounting for only a small fraction of all lung cancer cases. We describe a middle-aged woman who came to the hospital with cough and breathlessness, and imaging revealed multiple lung masses. A biopsy showed spindle-shaped tumor cells positive for smooth muscle markers, confirming leiomyosarcoma. She was treated with combination chemotherapy followed by oral pazopanib, showing good clinical and radiological improvement. This case highlights how rare and difficult to diagnose this tumor can be and the importance of tissue diagnosis in guiding effective treatment.

## Introduction

Primary pulmonary leiomyosarcomas (PPLs), a rare group of sarcomatous lung tumors, constitute less than 0.5% of the lung’s primary malignant neoplasms [1]. Patients with PPLs may be asymptomatic, and most tumors are incidentally detected. The tumors seem to originate from the smooth muscle cells of the bronchial structures and pulmonary blood vessels [2]. A five-year survival rate of 60% has been documented in previous studies [3]. Early detection and complete surgical resection of the tumors are associated with a better prognosis.

In this report, we describe a middle-aged woman with pulmonary mass lesions, subsequently diagnosed as having pulmonary leiomyosarcoma. This case stands out due to its rarity and atypical presentation, as PPLs usually appear as a solitary mass, whereas our patient presented with multiple bilateral lung nodules, mimicking metastatic disease.

## Case presentation

A 47-year-old female presented to the Outpatient Department with complaints of breathlessness and a non-productive cough for a month. There was no history of constitutional symptoms. She did not give a history of tuberculosis or contact with persons having active tuberculosis infection. On examination, she was afebrile, tachypneic, tachycardic, normotensive, and maintaining optimal oxygen saturation on room air. Examination of other systems was non-contributory. Initial laboratory investigations included hemoglobin 9.2 g/dL (10.5-14.0), total leukocyte count 14750 (4.4-11.0), and platelets 5.92 lakh. Coagulation profile, basic renal function, and liver function tests were within the normal range (Table 1).

**Table 1:** Quantitative laboratory investigations

Test/Parameter	Result	Reference Range	Unit
Hemoglobin	9.2	12 - 15	g/dL
Total Leukocyte Count	14750	4000 - 11000	cells/mm^3^
Platelet Count	5.92	1.5 - 4.5	10^5^/μL
Serum Creatinine	0.4	0.5 - 0.9	mg/dL
Alanine Transaminase (ALT)	14	< 35	IU/L
Aspartate Transaminase (AST)	11	< 35	IU/L
Total Bilirubin	0.22	< 1.2	mg/dL

**Figure 1:**
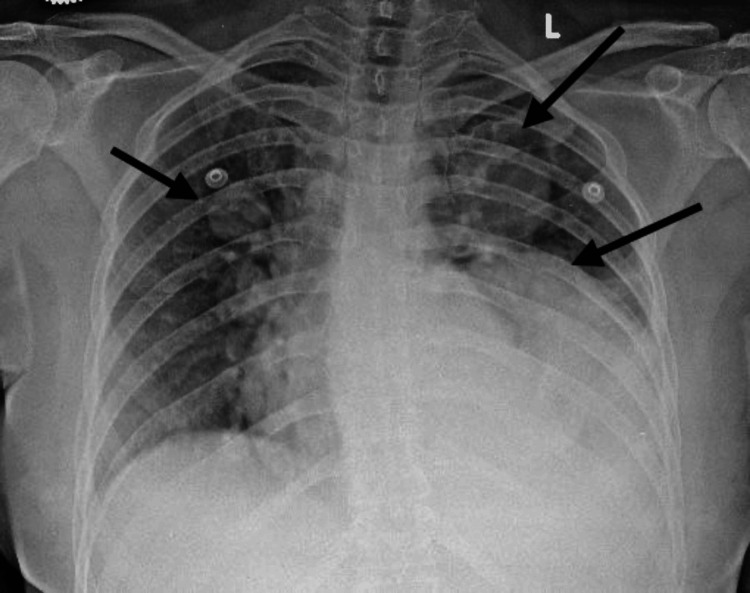
Chest radiograph showing bilateral well-defined, homogeneous opacities (arrows)

**Figure 2:**
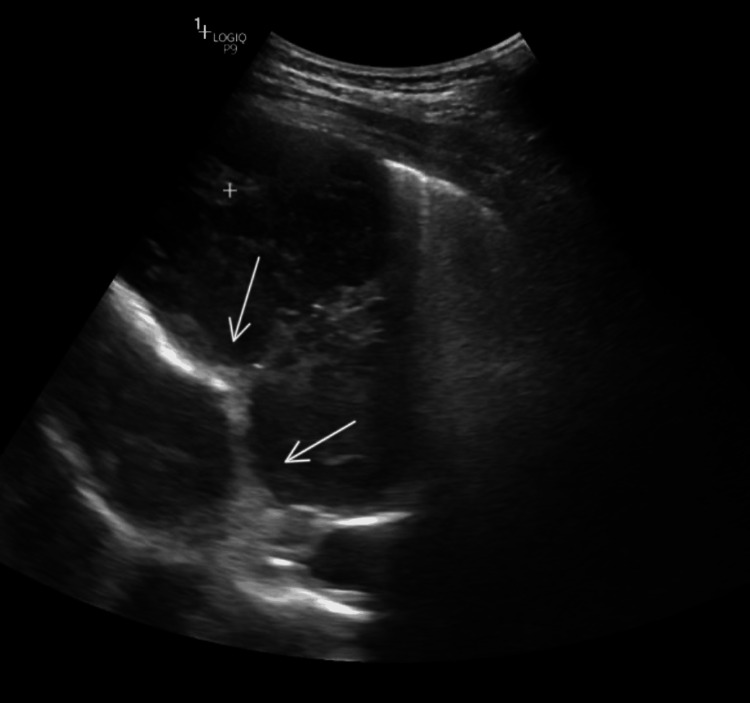
Ultrasonogram showing solid lesion with internal septations in the left lower lobe (arrows)

The chest radiograph showed bilateral middle- and left lower-zone, well-defined, rounded, homogeneous opacities (Figure 1). Ultrasonography was performed, which revealed a relatively well-defined solid lesion, measuring approximately 9.2 × 9.4 × 6 cm, with minimal internal vascularity and septations, in the left lower lobe of the lung (Figure 2).

**Figure 3:**
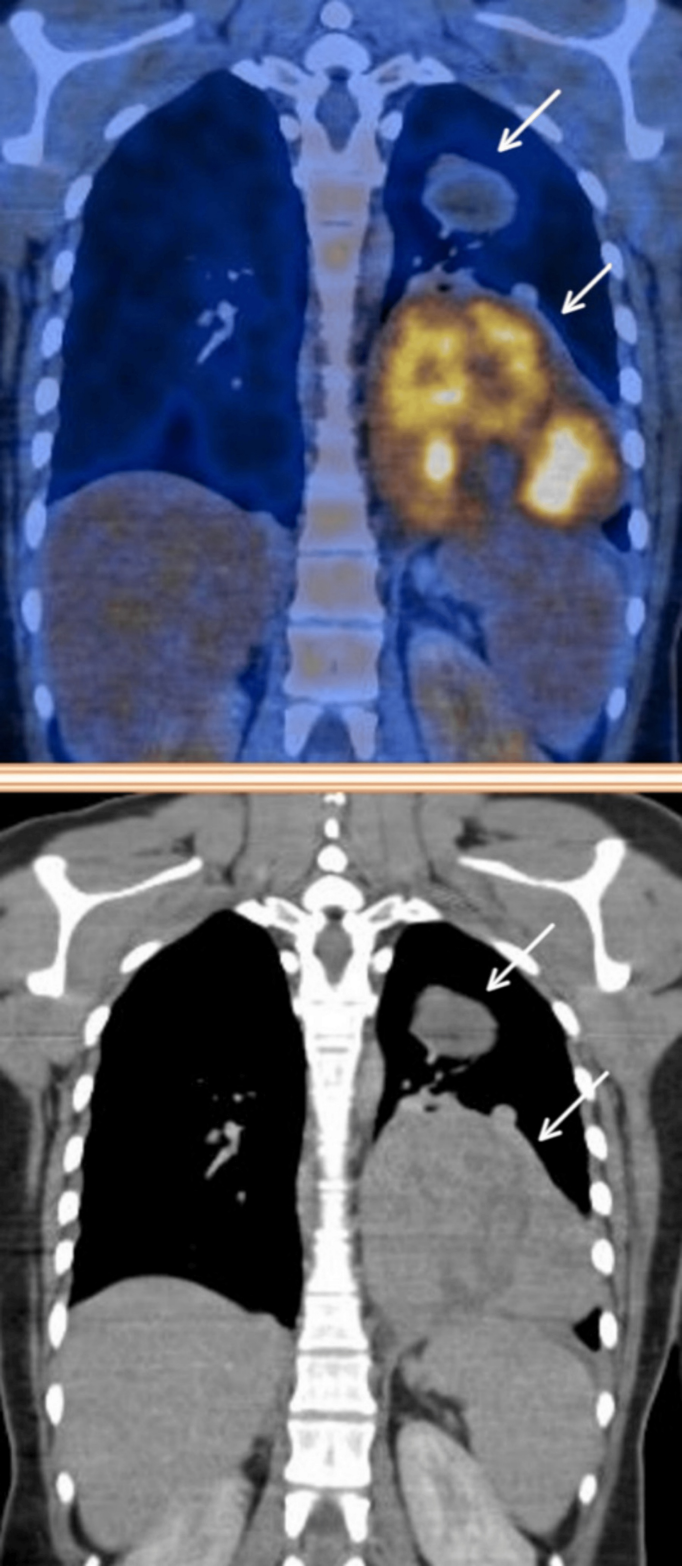
Positron emission tomography showing metabolically active lesions in the left lower lobe (arrows)

Subsequently, a positron emission tomography-computed tomography (PET-CT) scan was done, which showed metabolically active, irregular, lobulated, well-defined, heterogeneously enhancing lesions in the left lower lobe of the lung, measuring approximately 75 × 68 mm and 80 × 64 mm, with an SUV max of 8.5, along with multiple other metabolically active, randomly distributed, heterogeneously enhancing nodules in both lungs. Mediastinal lymph nodes, including prevascular, right paratracheal, and subcarinal nodes, showed faint metabolic activity. The uterus and bilateral ovaries were unremarkable, with no extrathoracic uptake elsewhere (Figure 3).

**Figure 4:**
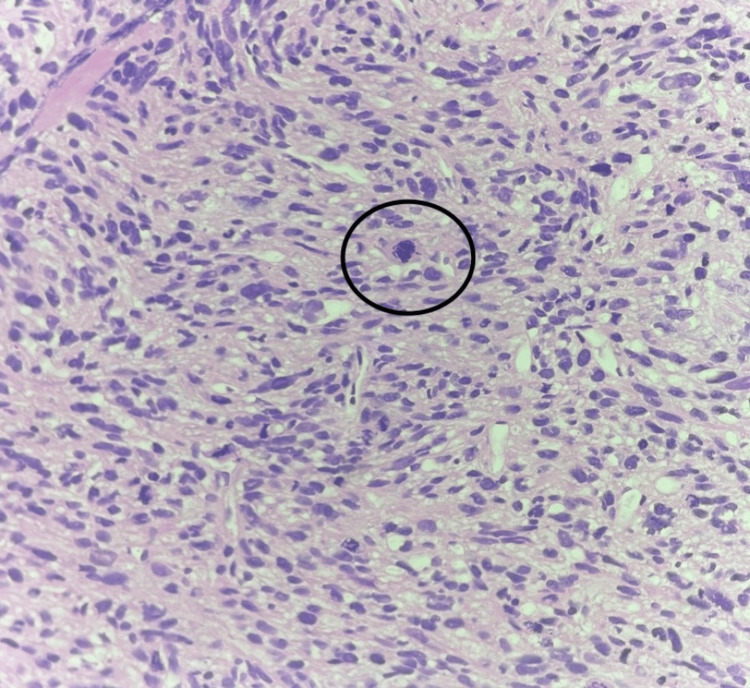
High-power field view showing features suggestive of leiomyosarcoma with tumor cells arranged in sweeping fascicles and mitotic figures highlighted

The patient underwent ultrasonographic transthoracic biopsy of the left lower lobe mass. Microscopic examination revealed cores of tumor tissue predominantly composed of spindle cells arranged in fascicles and interlacing bundles. The individual cells had moderate cytoplasm and oval to spindle-shaped vesicular nuclei. Increased mitotic figures, around 10 to 12 per high-power field, and atypical mitotic figures were noted. Focal areas of necrosis were also seen (Figure 4). 

**Figure 5:**
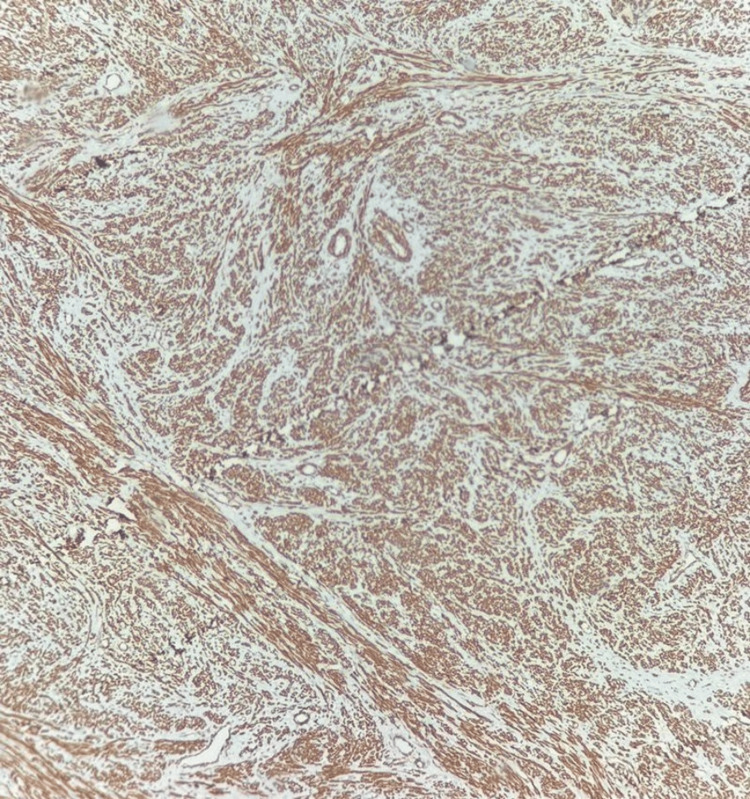
Smooth muscle actin immunopositive

**Figure 6:**
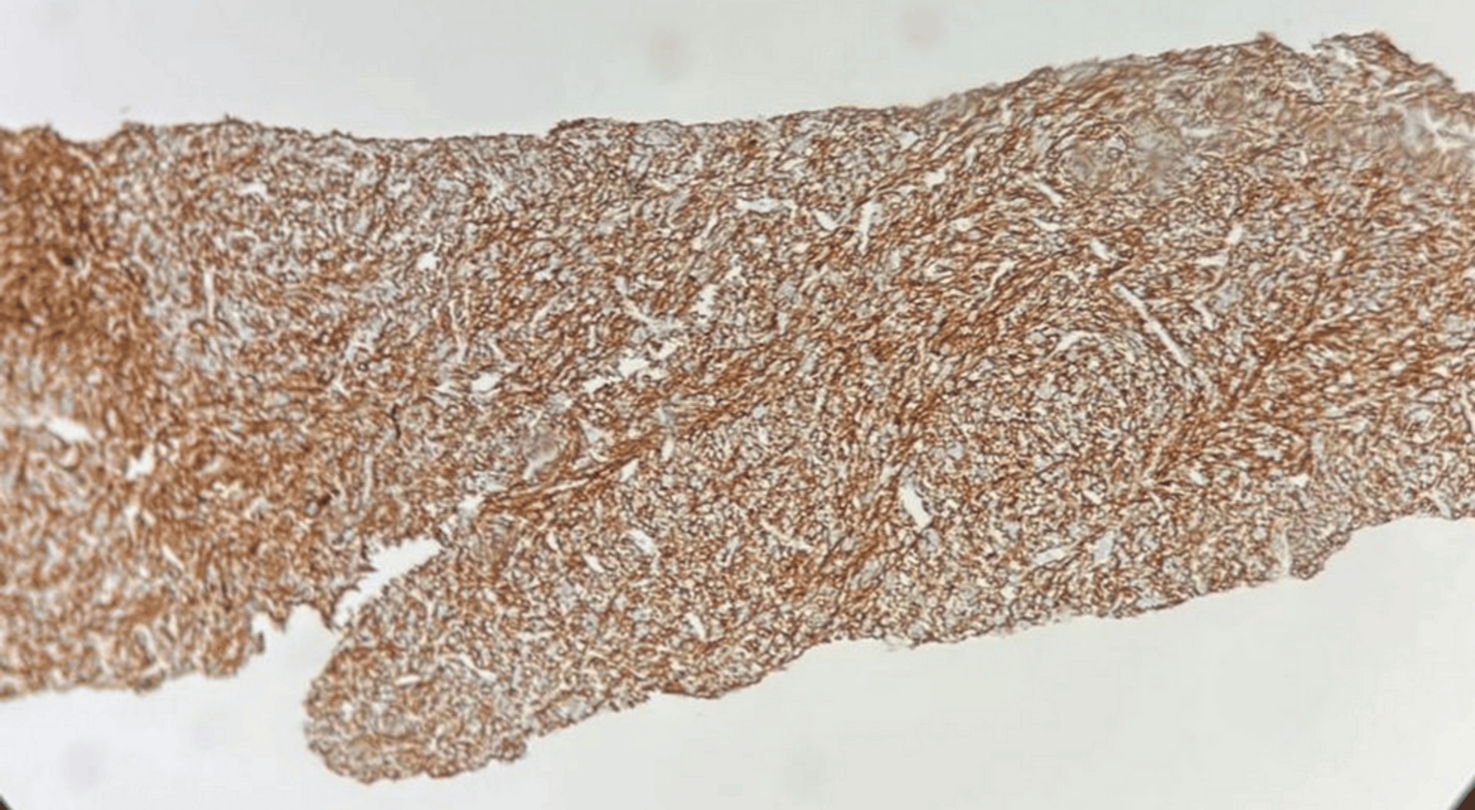
Desmin immunopositive

On immunohistochemistry, the tumor cells were immunopositive for smooth muscle actin (SMA) and desmin (Figures 5-6).

**Figure 7:**
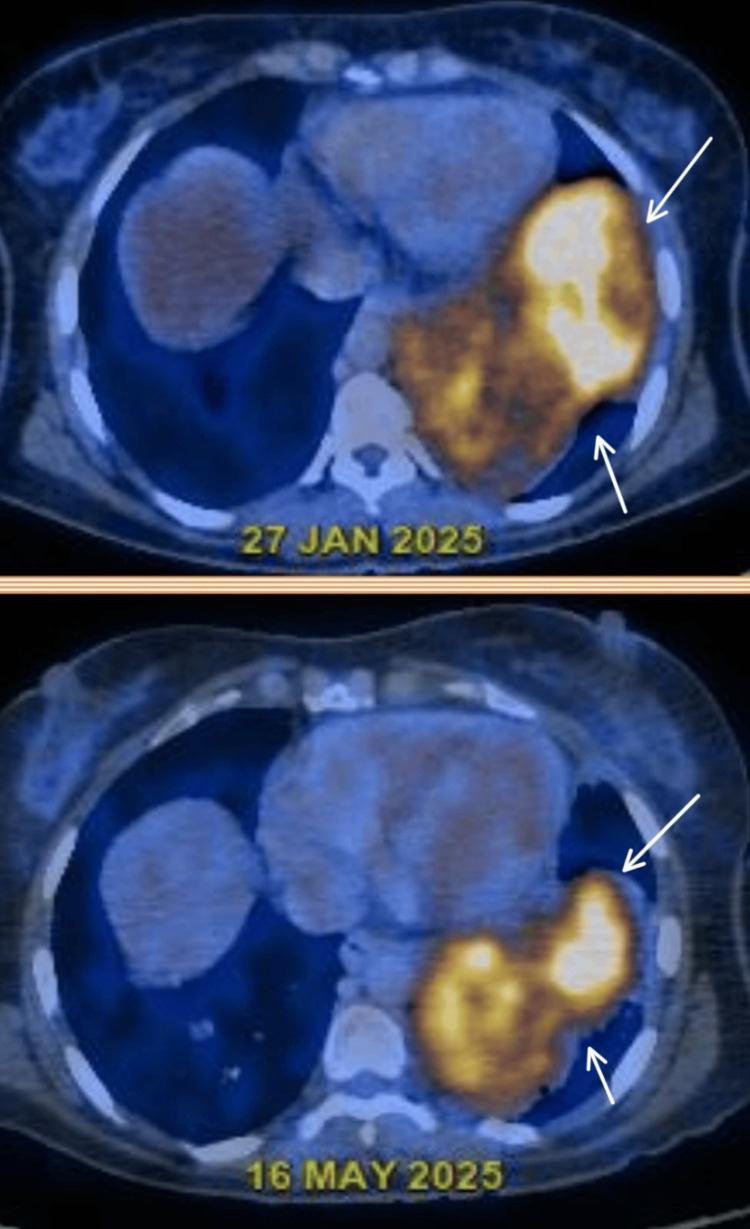
Follow-up positron emission tomography imaging showing a reduction in the size and metabolic activity of the lesion following chemotherapy (arrows)

The tumor cells were negative for myogenin, S100, and CD34. TLE1 and STAT6 were performed to rule out synovial sarcoma, and solitary fibrous tumors showed non-specific staining. Gastrointestinal stromal tumors were ruled out, as the tumor cells did not stain positive for DOG1 and CD117. The Ki-67 labeling index was found to be 60%. The mitotic count of the tumor was 10 to 12 per high-power field, pointing toward a diagnosis of Grade 2 leiomyosarcoma of intermediate grade, according to the French Federation of Cancer Centres Sarcoma Group (FNCLCC), exhibiting high proliferation and intermediate prognosis. On histopathological and immunohistochemical evaluation, the tumor showed features suggestive of leiomyosarcoma. The patient was subsequently referred to the Oncology Department for further management. She was initiated on combination chemotherapy with doxorubicin and ifosfamide. A follow-up PET-CT scan performed after completion of the regimen demonstrated a reduction in both the size and metabolic activity of the primary lesion. In view of this favorable response, she was transitioned to maintenance therapy with oral pazopanib (Figure 7).

## Discussion

PPL represents the most frequent histopathological subtype among primary pulmonary sarcomas; however, it remains an exceedingly rare entity, accounting for only 0.5% of all primary lung tumors and showing a reported male-to-female ratio of approximately 2.5:1 [4].

These tumors can arise in various anatomical locations within the respiratory system - endobronchially (20%), intraparenchymally (70%), or within the pulmonary artery (10%) [5]. Tumor extension into adjacent lung parenchyma, bronchi, or regional lymph nodes occurs in up to 50% of cases, while distant metastases are observed in approximately 20% [6]. Molecular mechanisms of tumorigenesis include loss of tumor suppressor genes, dysregulation of mTOR signaling pathways, upregulation of growth and angiogenic factors, chromosomal instability, and epigenetic alterations.

Clinically, PPLs often mimic bronchogenic carcinoma, presenting with non-specific respiratory symptoms such as cough, dyspnea, hemoptysis, sputum production, chest pain, and unintentional weight loss [7]. Radiologically, the findings are equally variable and non-specific, ranging from collapse, consolidation, or solitary mass lesions to bilateral nodular opacities and patchy infiltrates, occasionally accompanied by hilar or vascular prominence [8].

Histopathology and immunohistochemistry form the cornerstone of diagnosis. Microscopically, pulmonary leiomyosarcomas exhibit fascicles of spindle-shaped cells arranged in intersecting bundles, with elongated, blunt-ended nuclei and eosinophilic cytoplasm. Features such as nuclear pleomorphism, atypical mitotic figures, necrosis, and vascular invasion are often evident, particularly in high-grade lesions.

On immunohistochemistry, PPLs typically express smooth muscle markers, including SMA, desmin, and h-caldesmon, confirming smooth muscle differentiation. They are characteristically negative for epithelial markers (cytokeratins), neural markers (S-100), and c-KIT (CD117), helping to distinguish them from other spindle-cell neoplasms. A high Ki-67 proliferation index is frequently observed, reflecting the tumor’s aggressive biological behavior.

While pulmonary leiomyosarcoma can occur as a metastatic lesion, true PPLs are distinctly uncommon. Hence, imaging of the uterus and bilateral ovaries is essential to exclude a uterine primary, given that uterine leiomyosarcoma is a common source of pulmonary metastases. Metastatic dissemination occurs primarily through hematogenous and lymphatic routes, though lymph node involvement remains an unusual feature in pulmonary leiomyosarcoma [9,10].

Surgical resection remains the mainstay of treatment and offers the best chance for long-term survival. Complete surgical excision of a small, well-differentiated tumor can be potentially curative, with reported five-year survival rates between 29% and 40%. Radiotherapy is generally reserved for palliative purposes, particularly in cases of incomplete resection or unresectable disease. Chemotherapy, most commonly with doxorubicin- or ifosfamide-based regimens, is indicated for advanced, metastatic, or bilateral disease, as well as for patients with extrathoracic spread [11]. 

## Conclusions

PPL is a rare and often unexpected diagnosis, as its symptoms and imaging findings closely resemble those of more common lung cancers. Because of this, diagnosis can be challenging and usually depends on careful histopathological and immunohistochemical evaluation. It is also essential to rule out other primary sites, especially the uterus, before confirming a pulmonary origin.

Early detection and complete surgical removal offer the best chance of long-term survival. In advanced or metastatic cases, chemotherapy and newer targeted drugs, like pazopanib, can help control disease progression. This case underscores the need for clinicians to keep rare tumors, like pulmonary leiomyosarcoma, in mind when evaluating lung masses, and highlights the value of a multidisciplinary approach in diagnosis and treatment.

## References

[1] Attanoos RL, Appleton MA, Gibbs AR (1996). Primary sarcomas of the lung: a clinicopathological and immunohistochemical study of 14 cases. Histopathology.

[2] Xie X, Chen Y, Ding C (2016). Primary pulmonary leiomyosarcoma: a case report. Oncol Lett.

[3] Janssen JP, Mulder JJ, Wagenaar SS, Elbers HR, van den Bosch JM (1994). Primary sarcoma of the lung: a clinical study with long‑term follow‑up. Ann Thorac Surg.

[4] Cordes BG, Collins BT, McDonald JW, Khosla A, Salimi Z (1999). Fine needle aspiration biopsy of primary leiomyosarcoma arising from a pulmonary vein. Acta Cytol.

[5] Sardenberg RA, Cangnaci Neto R, Cavalcanti F, Younes RN (2011). High-grade primary pulmonary leiomyosarcoma. Einstein (Sao Paulo).

[6] Kim JH, Gutierrez FR, Lee EY, Semenkovich J, Bae KT, Ylagan LR (2003). Primary leiomyosarcoma of the pulmonary artery: a diagnostic dilemma. Clin Imaging.

[7] Demirci NY, Naurzvai N, Kirbaş I, Akyürek N, Gürsel G, Öztürk C (2018). Pulmonary artery leiomyosarcoma: a clinical dilemma. Lung India.

[8] Dixit R, Banerjee A, Panjabi M (2007). Primary leiomyosarcoma of the lung. Lung India.

[9] Nath D, Arava S, Joshi P, Madan K, Mathur S (2015). Primary pulmonary leiomyosarcoma of lung: an unusual entity with brief review. Indian J Pathol Microbiol.

[10] Yellin A, Rosenman Y, Lieberman Y (1984). Review of smooth muscle tumours of the lower respiratory tract. Br J Dis Chest.

[11] Moran CA, Suster S, Abbondanzo SL, Koss MN (1997). Primary leiomyosarcomas of the lung: a clinicopathologic and immunohistochemical study of 18 cases. Mod Pathol.

